# Data concerning AED registration in the Danish AED Network, and cardiac arrest-related characteristics of OHCAs, including AED coverage and AED accessibility

**DOI:** 10.1016/j.dib.2019.103960

**Published:** 2019-04-29

**Authors:** Lena Karlsson, Carolina Malta Hansen, Mads Wissenberg, Steen Møller Hansen, Freddy K. Lippert, Shahzleen Rajan, Kristian Kragholm, Sidsel G. Møller, Kathrine Bach Søndergaard, Gunnar H. Gislason, Christian Torp-Pedersen, Fredrik Folke

**Affiliations:** a Department of Cardiology, Copenhagen University Hospital Gentofte, Hellerup, Denmark; b Emergency Medical Services Copenhagen, University of Copenhagen, Denmark; c Department of Cardiology, Nephrology, and Endocrinology, Copenhagen University Hospital Hillerød, The Region of Northern Zealand, Denmark; d Unit of Epidemiology and Biostatistics, Aalborg University Hospital, Aalborg, Denmark; e Department of Cardiology, Aalborg University Hospital, Aalborg, Denmark; f The National Institute of Public Health, University of Southern Denmark, Copenhagen Denmark; g The Department of Health Science and Technology, Aalborg University, Aalborg, Denmark

**Keywords:** Cardiac arrest, Resuscitation, Automated external defibrillator, Survival

## Abstract

The data presented in this article is supplemental data related to the research article entitled “Automated external defibrillator accessibility is crucial for bystander defibrillation and survival: a registry-based study” (Karlsson et al., 2019). We present detailed data concerning: 1) the type of location for deployed and registered automated external defibrillators (AEDs) in the nationwide Danish AED Network; 2) the number of registered AEDs in the nationwide Danish AED Network, and changes in AED registration (according to year and type of AED location); 3) the number of AEDs being withdrawn from the AED network between the years 2007–2016. We also report data on baseline cardiac arrest-related characteristics of out-of-hospital cardiac arrests (OHCAs) that occurred in Copenhagen, Denmark, between 2008 and 2016. Cardiac arrest-related characteristics are further described according to AED accessibility (accessible vs. inaccessible AED at the time of OHCA) for OHCAs covered by an AED (AED ≤200 m route distance of an OHCA). Finally, we report data on distance to the nearest accessible AED for bystander defibrillated OHCAs covered by an AED ≤200 m route distance where the AED was inaccessible at the time of OHCA.

**Table:** Specifications table

Subject area	Public access defibrillation.
More specific subject area	AED registration, location and accessibility of AEDs in cardiac arrests.
Type of data	Tables.
How data was acquired	Retrospective from the Danish AED Network, and the Emergency Medical Services in Copenhagen, Denmark.
Data format	Descriptive and analysed.
Experimental factors	Data on registered AEDs within the nationwide Danish AED Network was used to categories AEDs according to type of AED location for placement and analysed according to 1) the total number of AEDs withdrawn between 2007 and 2016, and 2) year of registration. Data on OHCAs that occurred in the city of Copenhagen was analysed to investigate 1) associations between cardiac arrest-related characteristics and whether an AED ≤200 m route distance was accessible or not at the time of OHCA, and 2) distances to the nearest accessible AED among bystander defibrillated OHCAs where the nearest AED ≤200 m route distance was inaccessible at the time of OHCA. Statistical analyses performed in SAS (software version 9.4, SAS institute Inc., NC, USA), and distance calculations with the network analyst feature in ArcMap 10.5 [2].
Experimental features	Registry-based, cohort study.
Data source location	Copenhagen, Denmark.
Data accessibility	Data available in the present data article and the main article [1].
Related research article	Karlsson et al. Automated external defibrillator accessibility is crucial for bystander defibrillation and survival: a registry-based study. Resuscitation, 2019 [1].

**Table:** 

**Value of the data** • The extensive information provided on classification of type of AED location in a nationwide AED registry can serve as a benchmark for other countries and communities enabling comparison between AED registries internationally. • Data on temporal changes in AED registration within an AED network according to type of location can set the basis for new initiatives to improve AED use within communities. • The given data provides information of baseline characteristics among OHCAs occurring after implementation of an AED network. • The data provides information of associations between cardiac arrest-related characteristics and whether an AED ≤200 m was accessible or not at the time of OHCA. • The data provides information regarding longer distances to an accessible AED where the AED ≤200 m of the OHCA victim was inaccessible at the time of OHCA but the OHCA patient was bystander defibrillated.

## 1 Data

The data presented in this article is supplemental data to the study on AED accessibility and associations with bystander defibrillation and 30-day survival [1].

Table 1 describes the classification of AED location type for deployed and registered AEDs in the nationwide Danish AED Network. Table 2 describes newly registered AEDs in the Danish AED Network according to the year of registration and AED location type (2007–2016). Table 3 reports the number of AEDs withdrawn from the Danish AED Network, including type of AED location (2007–2016). Table 4 shows the cardiac arrest-related characteristics of the OHCA population in Copenhagen, Denmark, (2008–2016). Table 5 shows differences in cardiac arrest-related characteristics between OHCAs covered by an AED ≤200 m route distance and whether the AED was accessible or not at the time of OHCA. Table 6 reports within which route distances the nearest accessible AED was located for bystander defibrillated OHCAs that were covered by an inaccessible AED ≤200 m route distance.

**Table 1:** Specific types of locations for AEDs deployed and registered with the nationwide Danish AED Network.

AED location
***Companies/offices***
***School/education facilities*** (elementary and intermediate schools, universities, and other education facilities and libraries)
***Sports facilities*** (sports facility/centres, fitness centres, public swimming pool)
***Shopping malls/shops/banks*** (shopping malls/centres, grocery stores, banks, pharmacies)
***Unions/associations***
***Attractions/recreational areas*** (fair, playground, summer housing area, parks, golf courses, harbour)
***Residential settings*** (private home, nursing home, elderly housing/activity centre, housing association, apartments, housing support)
***Health clinics*** (general and dental practitioners, public and private hospitals)
***Public buildings***
***Church/community centre***
***Hotels and conference venues*** (including restaurants)
***Transportation facilities*** (bus terminal, train station, airport)
***Other*** (e.g., retrieval plant, utility, waste management stations, fire/police station)

AED, automated external defibrillator.

**Table 2:** AEDs newly registered with the nationwide Danish AED Network, according to the year of registration and type of location.

Newly registered AEDs per year, n (% of all AEDs registered in 2007–2016)	Year of registration										Total, n (%)
	2007, n (%)	2008, n (%)	2009, n (%)	2010, n (%)	2011, n (%)	2012, n (%)	2013, n (%)	2014, n (%)	2015, n (%)	2016, n (%)	
Type of AED location	140 (0.8)	506 (3.0)	926 (5.4)	2228 (13.0)	1842 (10.8)	2152 (12.6)	2252 (13.2)	2258 (13.2)	2115 (12.4)	2687 (15.7)	17 106 (100.0)
Companies/offices	21 (15.0)	125 (24.7)	250 (27.0)	700 (31.4)	568 (30.8)	669 (31.1)	610 (27.1)	645 (28.6)	635 (30.0)	780 (29.0)	5003 (29.2)
School/education facility	10 (7.1)	41 (8.1)	95 (10.3)	345 (15.5)	225 (12.2)	254 (11.8)	315 (14.0)	261 (11.6)	277 (13.1)	294 (10.9)	2117 (12.4)
Sports facility	56 (40.0)	134 (26.5)	154 (16.6)	325 (14.6)	205 (11.1)	174 (8.1)	164 (7.3)	144 (6.4)	109 (5.2)	158 (5.9)	1623 (9.5)
Residential settings	2 (1.4)	12 (2.4)	24 (2.6)	66 (3.0)	70 (3.8)	144 (6.7)	172 (7.6)	224 (9.9)	289 (13.7)	515 (19.2)	1518 (8.9)
Shopping malls/shops/banks	0 (0.0)	21 (4.2)	68 (7.3)	127 (5.7)	129 (7.0)	151 (7.0)	252 (11.2)	238 (10.5)	77 (3.6)	173 (6.4)	1236 (7.2)
Union/association	0 (0.0)	17 (3.4)	72 (7.8)	87 (3.9)	115 (6.2)	128 (6.0)	190 (8.4)	164 (7.3)	184 (8.7)	69 (2.6)	1026 (6.0)
Attractions/recreational areas	16 (11.4)	58 (11.5)	73 (7.9)	147 (6.6)	104 (5.7)	140 (6.5)	107 (4.8)	104 (4.6)	110 (5.2)	161 (6.0)	1020 (6.0)
Health clinics	13 (9.3)	27 (5.3)	43 (4.6)	111 (5.0)	96 (5.2)	114 (5.3)	121 (5.4)	89 (3.9)	81 (3.8)	86 (3.2)	781 (4.6)
Public building	12 (8.6)	19 (3.8)	47 (5.1)	107 (4.8)	68 (3.7)	83 (3.9)	54 (2.4)	98 (4.3)	81 (3.8)	72 (2.7)	641 (3.7)
Church/community centre	1 (0.7)	0 (0.0)	6 (0.7)	26 (1.2)	37 (2.0)	79 (3.7)	65 (2.9)	106 (4.7)	106 (5.0)	171 (6.4)	597 (3.5)
Hotels and conference venues	0 (0.0)	11 (2.2)	19 (2.1)	41 (1.8)	41 (2.2)	35 (1.6)	44 (2.0)	40 (1.8)	31 (1.5)	49 (1.8)	311 (1.8)
Transportation facility	2 (1.4)	6 (1.2)	7 (0.8)	15 (0.7)	18 (1.0)	40 (1.9)	9 (0.4)	26 (1.2)	24 (1.1)	32 (1.2)	179 (1.1)
Other	7 (5.0)	35 (6.9)	68 (7.3)	131 (5.9)	166 (9.0)	141 (6.6)	149 (6.6)	119 (5.3)	111 (5.3)	127 (4.7)	1054 (6.2)

AED, automated external defibrillator.

**Table 3:** Withdrawn AEDs, according to the type of location, nationwide (2007–2016).

Total AEDs withdrawn, n (%)	1805 (100.0)
Companies/offices	592 (32.8)
School/education facility	221 (12.2)
Sports facility	220 (12.2)
Shopping malls/shops/banks	159 (8.8)
Other	124 (6.9)
Public building	92 (5.1)
Residential settings	92 (5.1)
Health clinics	84 (4.7)
Union/association	71 (3.9)
Attractions/recreational areas	73 (4.0)
Hotels and conference venues	36 (2.0)
Transportation facility	21 (1.2)
Church/community centre	20 (1.1)

In total, 17 106 AEDs were registered with the nationwide Danish AED Network from 2007 through 2016. Of these, 1805 (10.6%) AEDs were withdrawn during the study period.

AED, automated external defibrillator.

**Table 4:** Cardiac arrest-related characteristics of the OHCA study population in Copenhagen (2008–2016).

Total OHCAs, n (%)	2500 (100.0)
Median age, year (IQR)	70 (59–80)
Male, year (IQR)	67 (56–77)
Female, year (IQR)	75 (64–86)
Male, n (%)	1550 (62.6)
Public location, n (%)	621 (24.8)
Median EMS response time^a^, min (IQR)	5 (4–7)
Shockable heart rhythm, n (%)	607 (24.3)
Bystander witnessed arrest, n (%)	1412 (57.3)
Bystander CPR, n (%)	1192 (48.6)
Bystander defibrillation, n (%)	126 (5.0)
30-day survival^b^, n (%)	351 (14.6)

OHCA, out-of-hospital cardiac arrest; IQR, interquartile range; EMS, emergency medical service; CPR, cardiopulmonary resuscitation.

Number of missing: age (n = 45), sex (n = 25), response time (n = 33), bystander witnessed status (n = 35), bystander CPR (n = 45).

a Time from dispatch of vehicle to arrival at scene of cardiac arrest.

b 87 cardiac arrests excluded due to missing information on 30-day survival.

**Table 5:** Differences in cardiac arrest-related characteristics between OHCAs covered by an accessible vs. an inaccessible AED.

Total OHCAs, n (%)	OHCAs located ≤200 m of accessible AED, n (%)	OHCAs located ≤200 m of inaccessible AED, n (%)	P value
	276 (48.8)	290 (51.2)	
Median age, year (IQR)	66 (54–77)	69 (57–79)	0.14
Male, year (IQR)	64 (53–73)	66 (55–76)	0.38
Female, year (IQR)	73 (59–85)	77 (65–87)	0.30
Male, n (%)	185 (68.8)	199 (69.8)	0.79
Public location, n (%)	152 (55.1)	100 (34.5)	<0.001
Shockable heart rhythm, n (%)	97 (35.1)	72 (24.8)	0.007
Median EMS response time^a^, min (IQR)	5 (3–7)	5 (3–6)	0.12
Bystander witnessed arrest, n (%)	169 (62.1)	168 (59.2)	0.47
Bystander CPR, n (%)	198 (72.5)	155 (54.8)	<0.001

OHCA, out-of-hospital cardiac arrest; IQR, interquartile range; EMS, emergency medical service; CPR, cardiopulmonary resuscitation; AED, automated external defibrillator.

Number of missing: age (n = 21), sex (n = 12), response time (n = 6), bystander witnessed status (n = 10), bystander CPR (n = 10).

a Time from dispatch of vehicle to arrival at scene of cardiac arrest.

**Table 6:** Distance to the nearest accessible AED for bystander defibrillated OHCAs covered by an inaccessible AED ≤200 m.

	Bystander defibrillated OHCAs covered by an inaccessible AED ≤200 m	Nearest accessible AED within longer distances, n (%)			
		201–300 m, n (%)	301–400 m, n (%)	401–500 m, n (%)	>500 m, n (%)
All OHCAs, n	14	3 (21.4)	3 (21.4)	3 (21.4)	5 (35.7)
Bystander witnessed OHCAs with shockable heart rhythm, n	13	3 (23.1)	3 (23.1)	3 (23.1)	4 (30.8)

AED, automated external defibrillator; OHCA, out-of-hospital cardiac arrest.

## 2 Experimental design, materials, and methods

This data article includes information on (1) registered AEDs within the nationwide Danish AED Network (2007–2016), and (2) OHCAs in the city of Copenhagen, Denmark (2008–2016).

A description of the data collected from the Danish AED Network, and how the specific type of location for AEDs deployed and registered in the network was determined can be found in the related main research article [1]. In the present data article, the number of newly registered AEDs is described per year and type of AED location (2007–2016). The number of withdrawn AEDs between the same period in time is reported overall and according to type of AED location.

OHCAs included in this data article were OHCAs of presumed cardiac cause not witnessed by the emergency medical service (EMS), and with known location and addresses, known bystander defibrillation status, and calculated route distances to registered AEDs in Copenhagen, Denmark (the OHCA population in the related main article [1]). OHCAs were registered by the Copenhagen physician-manned mobile emergency care unit in the municipality of Copenhagen (2008–2016), a method used previously [3], [4]. Distance calculations were made using road/pedestrian routes from OHCAs to AEDs in the software ArcMap 10.5 (network analyst feature) [1], [2]. An AED was defined as covering an OHCA if the OHCA occurred ≤200 m route distance from an AED that had been deployed before the date of OHCA. AED accessibility was assessed for every OHCA-AED pair [1].

Categorical variables are presented as absolute numbers and percentages, and continuous variables as medians with interquartile range (IQR). Cardiac arrest-related characteristics were investigated according to whether the nearest AED ≤200 m route distance of the OHCA was accessible or not at the time of OHCA. Categorical variables were compared with the chi-square test, and continuous variables with the Kruskal-Wallis test. A 2-sided p-value <0.05 was considered significant. Analyses were performed using SAS (software version 9.4, SAS institute Inc., NC, USA).
